# Supplementary material for: Mechanism of small molecule inhibition of Plasmodium falciparum myosin A informs antimalarial drug design
Source: Nat Commun. 2023 Jun 12;14:3463. doi: 10.1038/s41467-023-38976-7 (PMC10261046; doi:10.1038/s41467-023-38976-7)
Supplement: Supplementary file 1 — Supplementary Information [file 41467_2023_38976_MOESM1_ESM.pdf]

Supplementary Information

Mechanism of small molecule inhibition of *Plasmodium falciparum* myosin A informs antimalarial drug design

Dihia Moussaoui[1,2,$], James P. Robblee[3,$], Julien Robert-Paganin[1,$], Daniel Auguin[1,4], Fabio Fisher[5], Patricia M. Fagnant[3], Jill E. Macfarlane[3], Julia Schaletzky[6], Eddie Wehri[6], Christoph Mueller-Dieckmann[2], Jake Baum[5,7], Kathleen M. Trybus[3,#], Anne Houdusse[1,#].

Affiliations

[1]Structural Motility, Institut Curie, Université Paris Sciences et Lettres, Sorbonne Université, CNRS UMR144, 75248 Paris, France

[2]Structural Biology group, European Synchrotron Radiation Facility. 71, Avenue des Martyrs, 38000 Grenoble, France. Current affiliation of DM.

[3]Department of Molecular Physiology & Biophysics, University of Vermont, Burlington, VT, USA.

[4]Laboratoire de Biologie des Ligneux et des Grandes Cultures (LBLGC), Université d'Orléans, INRAE, USC1328, Orléans, France.

[5]Department of Life Sciences, Imperial College London, Exhibition Road, South Kensington, London SW7 2AZ, UK.

[6]Center for Emerging and Neglected Diseases, Drug Discovery Center, Berkeley, CA, USA.

[7]School of Medical Sciences, Faculty of Medicine & Health, UNSW Sydney, Kensington, 2052, NSW, Australia.

[#] Correspondence and requests for materials should be addressed to K.M.T. (email: kathleen.trybus@uvm.edu or to A.H. (email: anne.houdusse@curie.fr)

[$] These authors contributed equally

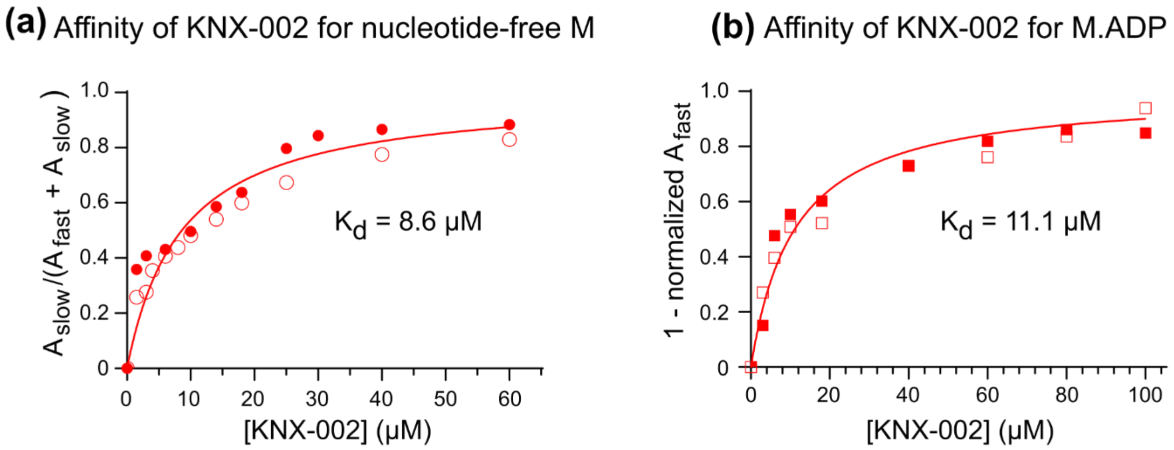

Supplementary Figure 1 *– Affinity of KNX-002 for nucleotide-free PfMyoA and PfMyoA.ADP*

(a) Affinity of KNX-002 for nucleotide-free PfMyoA (M). Fractional amplitude of the slow phase ($A_{slow}/(A_{fast} + A_{slow})$) as a function of KNX-002 concentration. 0.3 µM PfMyoA was pre-incubated with varying concentrations of KNX-002 and then rapidly mixed with either 3 µM mant-ATP (filled circles) or 6 µM mant-ADP (open circles) also containing KNX-002 (pre-mixing concentrations stated). Observed rate constants for the time courses were 6.5 ± 0.4 s^{-1} (no KNX-002) and 1.03 ± 0.01 s^{-1} (100 µM KNX-002) for mant-ATP and 12.7 ± 0.2 s^{-1} (no KNX-002) and 1.80 ± 0.02 s^{-1} (100 µM KNX-002) for mant-ATP. For intermediate KNX-002 concentrations, the fractional amplitude of the slow phase ($A_{slow}/(A_{fast} + A_{slow})$) of the biphasic transients is plotted against KNX-002 concentration. The K_d of KNX-002 for nucleotide-free PfMyoA is 8.6 µM (hyperbolic fit). The two resolvable kinetic phases suggest that the off rate of KNX-002 is very slow.

(b) Affinity of KNX-002 for PfMyoA in the presence of ADP (M.ADP). The disappearance of the fast phase (1 - (A_{fast} at measured [KNX-002] / A_{fast} in the absence of KNX-002)) as a function of KNX-002 concentration. 0.3 µM PfMyoA was pre-incubated with 100 µM ADP and the indicated amount of KNX-002 and then rapidly mixed with 3 µM mant-ATP also containing the indicated amount of KNX-002. The K_d of KNX-002 for PfMyoA.ADP is 11.1 µM (hyperbolic fit). All time courses were biphasic with a fast phase rate of 1.44 ± 0.03 s^{-1} of varying amplitude and a slow phase having a constant rate (0.08 ± 0.01 s^{-1}) and constant amplitude.

In a separate experiment, 200 µM KNX-002 was mixed with PfMyoA.mant-ADP. A small increase in bound mant-ADP fluorescence (excited via a Trp residue in PfMyoA) was observed at a rate of ~1 s^{-1}. This observation is consistent with the ability of KNX-002 to bind to PfMyoA that has mant-ADP bound at the active site.

Conditions: 10 mM HEPES pH 7.5, 50 mM KCl, 4 mM $MgCl_2$, 1 mM EGTA, 1 mM DTT, 1% DMSO, 30°C. Two independent experiments were performed. Source data are provided as a Source Data file.

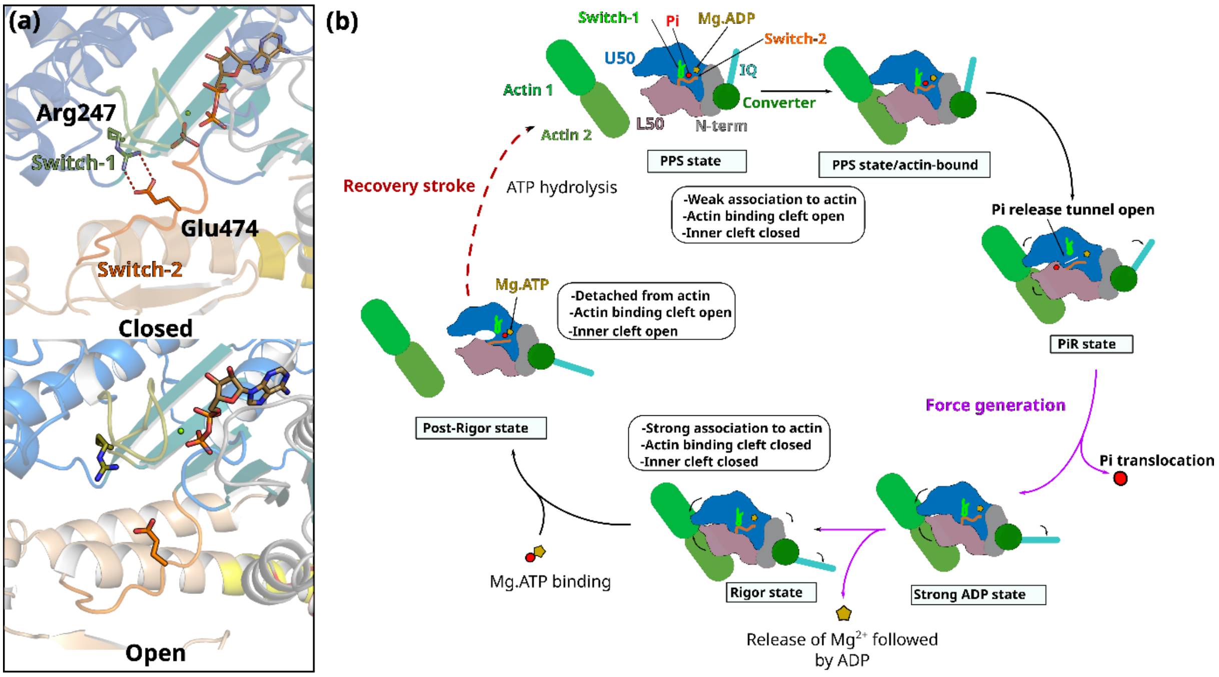

<u>Supplementary Figure 2</u> *– <u>Backdoor and Schematic motor cycle of a myosin</u>*.

(a) Backdoor of myosins has a fully closed configuration in the Pre-powerstroke state, but not in the post-Rigor state.

(b) Motor cycle - In the post-rigor (PR) state, the myosin is bound to ATP, with the lever arm down and the actin binding cleft open, including the inner cleft. The PR is detached from actin. Priming of the lever arm occurs during the recovery stroke, resulting in the pre-power stroke state (PPS) in which ATP hydrolysis is facilitated thanks to the specific positioning of Switch-2 in this state (inner cleft or back-door closed). The PPS state can associate weakly to the actin track. This association triggers the transition towards the P_i release state (P_iR) that allows P_i release. It is presumed that a small movement of the lever arm is sufficient to rearrange the actin-binding site in the P_iR state so that an opening of the P_i release tunnel would then allow the P_i to escape the active site[1]. The P_iR state initiates force production. A large lever arm swing and cleft closure drive the transition to the Strong ADP state during the powerstroke. Finally, the release of the Mg^{2+} ion followed by ADP occurs after the transition to the Rigor state. The Rigor state differs from the Strong ADP state by a further small lever arm swing. In the Rigor state, myosin is ready to bind a new ATP molecule. This leads to fast detachment from actin while myosin adopts a post-rigor state and starts a new cycle. The structural features underlying myosin force production are reviewed in[2].

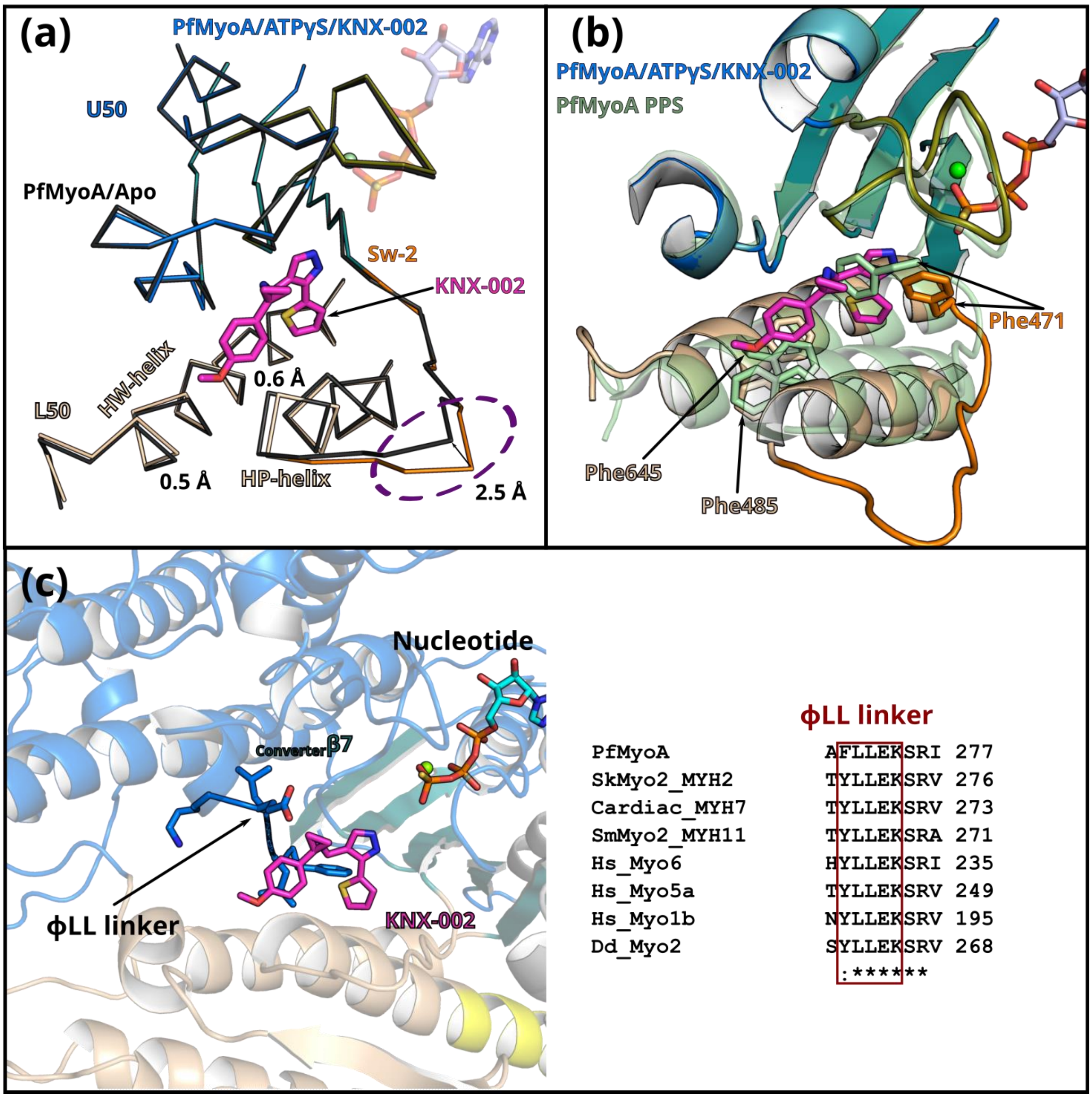

<u>Supplementary Figure 3</u> *<u>– Analysis of the binding pocket of KNX-002</u>.* **(a)** Superimposition on the U50 subdomain of PfMyoA bound to KNX-002 (colored by subdomains, PfMyoA/ATPγS/KNX-002) and PfMyoA in the apo condition (black, PfMyoA/ATPγS/Apo). Small local accommodations are induced in the pocket by the binding of the drug, the most remarkable being a shift in Switch-2 (Sw-2) position (indicated with a dashed purple circle). **(b)** Superimposition of PfMyoA/ATPγS/KNX-002 and PfMyoA in the pre-powerstroke (PPS) state (PDB code 6YCX,[3]) on the U50 subdomain. The superimposition shows that KNX-002 cannot bind in PPS. In particular, a clash would occur if Switch-2 rearranges to close the backdoor as found in PPS. Note in particular the position F471 adopts in the PPS state. **(c)** Cartoon representation showing the ΦLL linker, a conserved element located in the U50 after the $_{\text{Transducer}}$β7 strand. Alignment of myosin sequences show the conservation of the ΦLL linker: PfMyoA; human fast skeletal muscle myosin 2 (SkMyo2_MYH2); human β-cardiac muscle myosin 2 (Cardiac_MYH7); human smooth muscle myosin 2 (SmMyo2_MYH11); human myosin 6 (Hs_Myo6); human myosin 5a (Hs_Myo5a); human myosin-1b (Hs_Myo1b); *Dictyostelium discoideum* myosin 2 (DdMyo2).

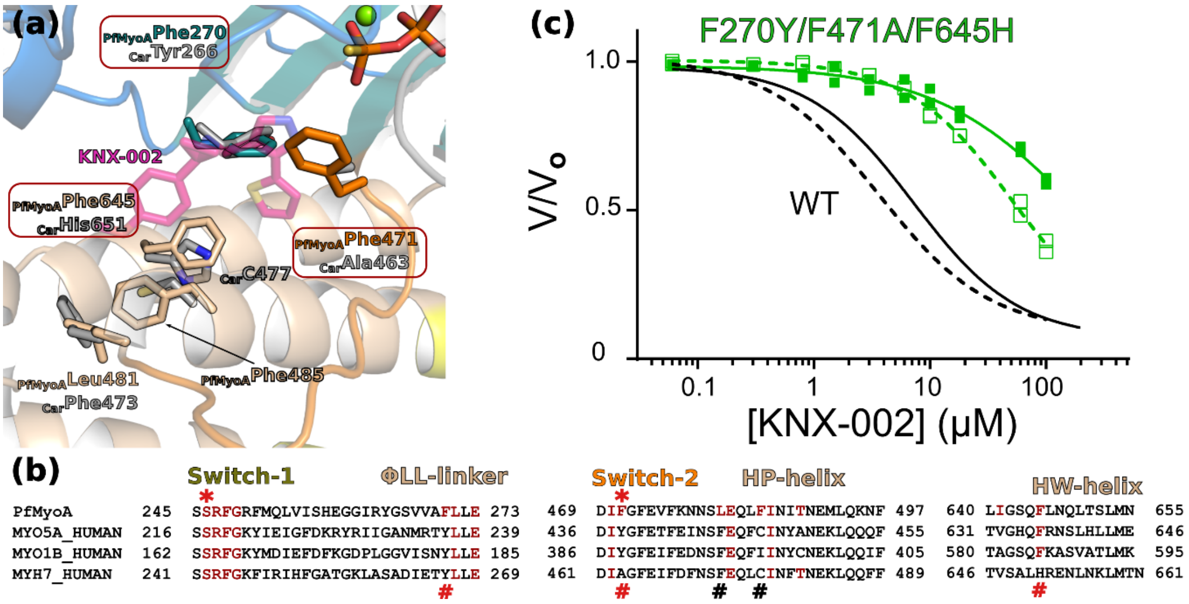

***Supplementary Figure 4** – Validation of the KNX-002 binding pocket.* **(a)** Representation of the differences existing in the KNX-002 binding pocket in PfMyoA and in β-cardiac myosin in the PR state (PDB code 6FSA,[4]). The two structures are aligned on the U50 subdomain of PfMyoA. The sequence differences in cardiac are represented as stick in grey (for detail see Fig. 3f). Residues mutated in PfMyoA to alter the pocket and prevent inhibition are represented in a red box. **(b)** Sequence alignment of the different structural elements of KNX-002 binding pocket in several myosins: *Plasmodium falciparum* myosin A (PfMyoA); *Homo sapiens* myosin 5a (MYO5A_HUMAN); *Homo sapiens* myosin 1b (MYO1B_HUMAN); *Homo sapiens* β-cardiac myosin (MYH7_HUMAN). Residues involved in polar contacts are labelled with a red star; positions of important differences are labelled with a hash, the three mutations in the triple mutant F270Y/F471A/F645H are labelled with a red hash. **(c)** KNX-002 poorly inhibits the actin-activated (filled green squares, IC_{50}>100 µM) and the basal ATPase activity (open green squares, 52 µM of the triple mutant F270Y/F471A/F645H compared to its effect on WT PfMyoA (WT fits reproduced from Fig. 1a, actin-activated (solid line) and basal (dashed black line) ATPase activity). n=2 for each assay. Source data are provided as a Source Data file.

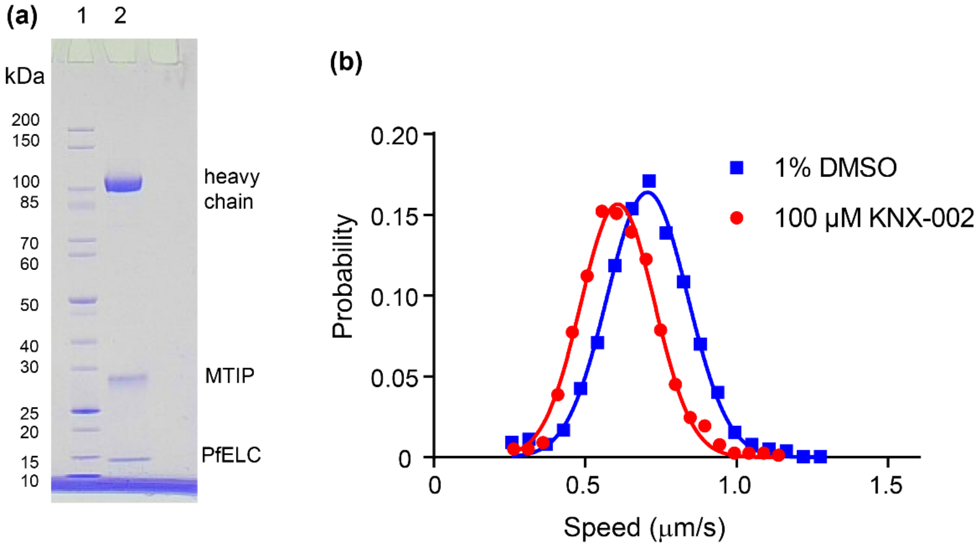

<u>Supplementary Figure 5</u> *<u>– Characterization of the triple mutant F270Y/F471A/F645H</u>.*

(a) Coomassie stained 4-12% SDS-PAGE gel of (lane 1) molecular mass markers and (lane 2) the F270Y/F471A/F645H triple mutant. The uncropped gel image is shown in Supplementary Figure 11. **(b)** Speed distributions from a representative *in vitro* motility assay of the F270Y/F471A/F645H triple mutant. 1% DMSO, 0.71 ± 0.13 µm/s; 100 µM KNX-002, 0.61 ± 0.12 µm/s. Values reported are mean ± SD. 799 filaments counted (n=8 movies) for 1% DMSO and 775 filaments (n=6 movies) counted for 100 µM KNX-002. Conditions: 25 mM imidazole pH 7.5, 150 mM KCl, 4 mM $MgCl_2$, 1 mM EGTA, 10 mM DTT, 2 mM MgATP, 0.5% methylcellulose, 1% DMSO, 30°C. The speeds in the presence or absence of KNX-002 are statistically insignificant using a two-sided z-test (p = 0.572). Source data are provided as a Source Data file.

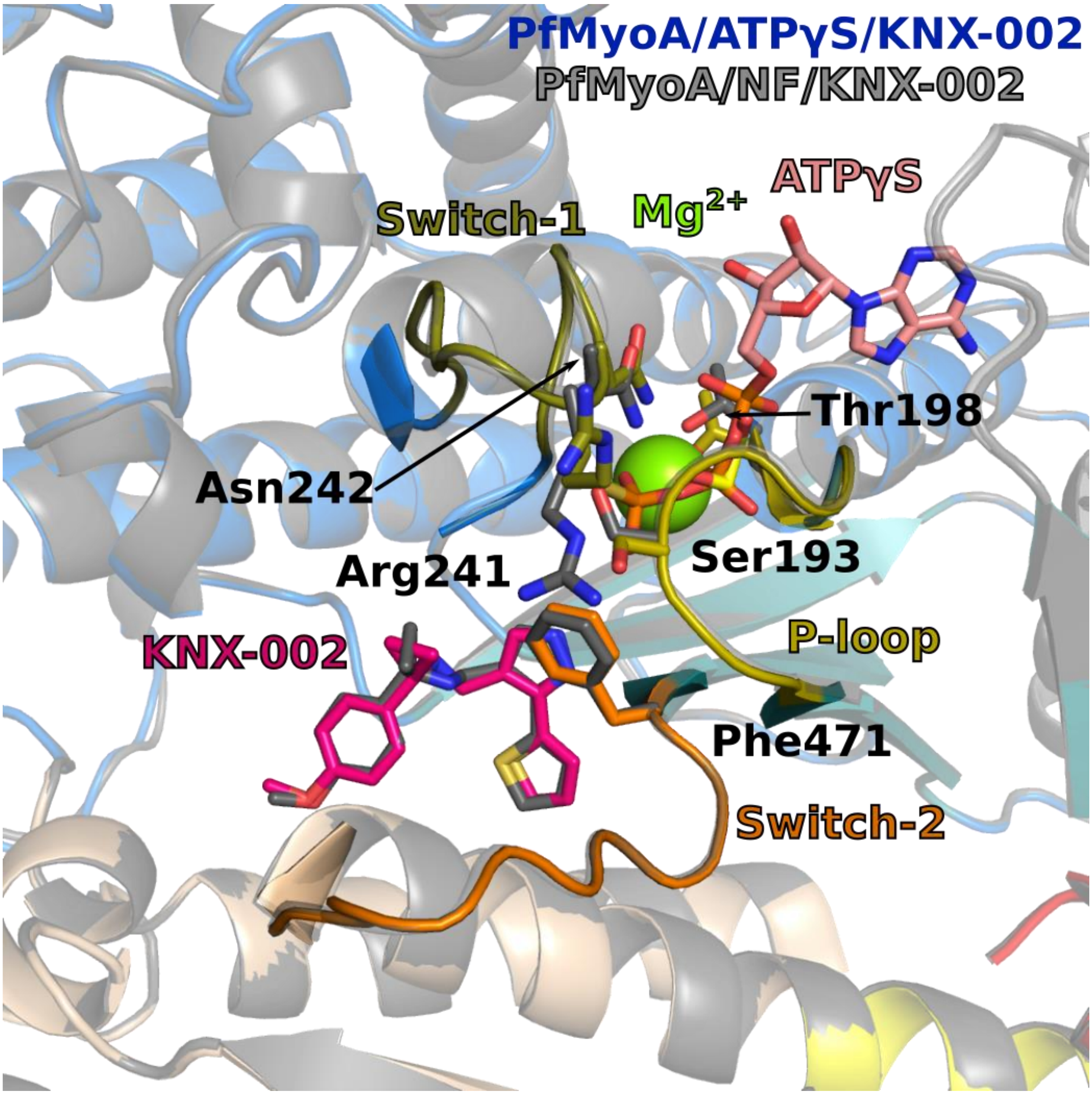

<u>Supplementary Figure 6</u> *– <u>Comparison of the structures PfMyoA/ATPγS/KNX-002 and PfMyoA/NF/KNX-002</u>. The structures of PfMyoA complexed to ATPγS and KNX-002 (PfMyoA/ATPγS/KNX-002, colored by subdomain) and PfMyoA complexed to KNX-002 in the nucleotide free state (PfMyoA/NF/KNX-002, dark grey). In the presence of KNX-002, the structures of PfMyoA are highly similar independent of the presence of the nucleotide. The only differences are in the local conformation of residues located in the P-loop (dark yellow) and Switch-1 (olive green); the side chains of these residues are represented as sticks. The two structures are superimposed on the U50 and L50 (residues 182-667).*

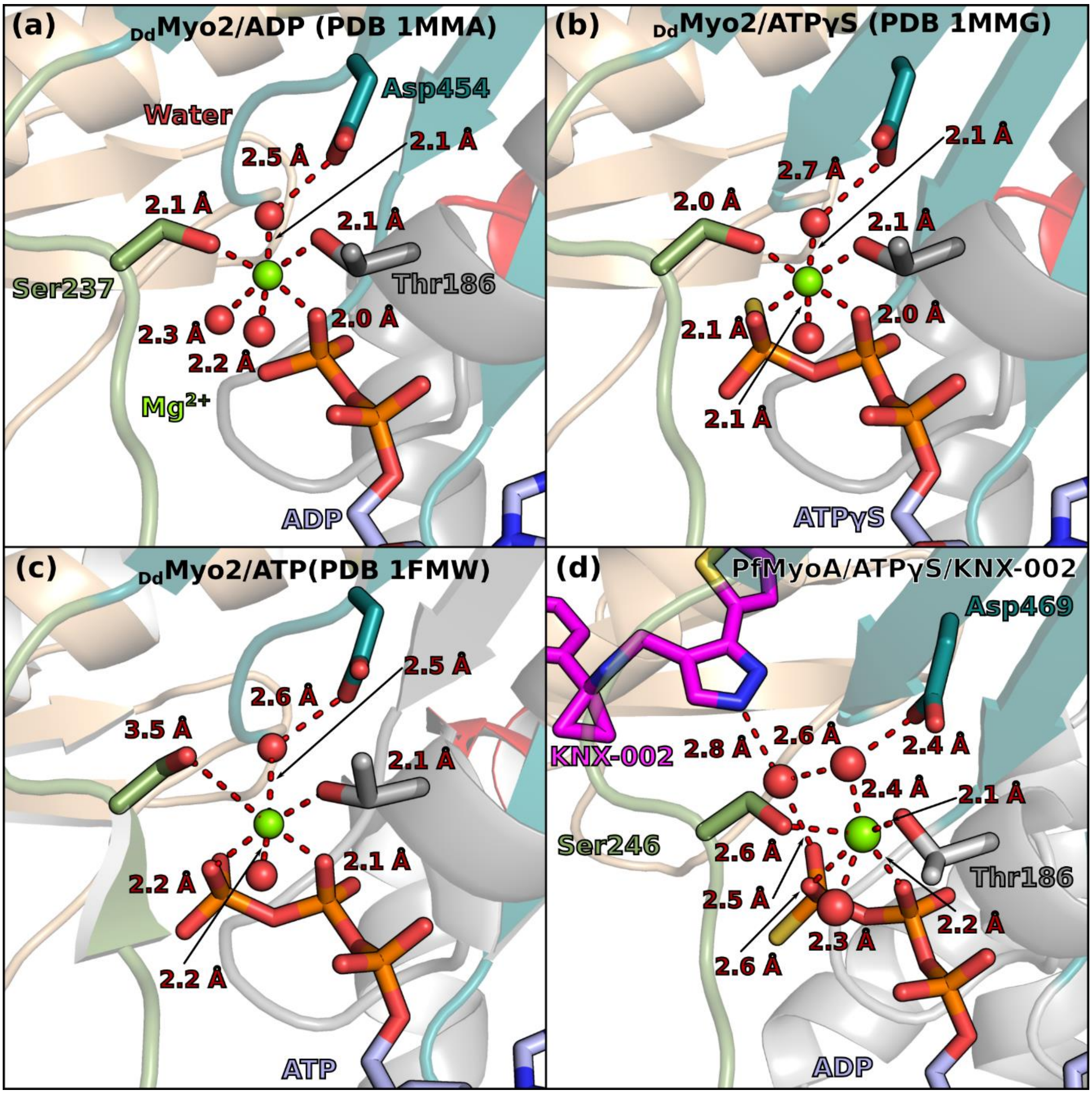

Supplementary Figure 7 *– The presence of γ-phosphate does not alter the coordination of Mg^{2+} ion.* Comparison of Mg^{2+} coordination in DdMyo2 structure complexed to **(a)** ADP (PDB code 1MMA)[5], **(b)** DdMyo2 complexed to ATPγS (PDB code 1MMG)[5], **(c)** DdMyo2 structure complexed to ATP (PDB code 1FMW)[6]. **(d)** For comparison, the coordination of PfMyoA complexed to ATPγS and KNX-002 (PfMyoA/ATPγS/KNX-002).

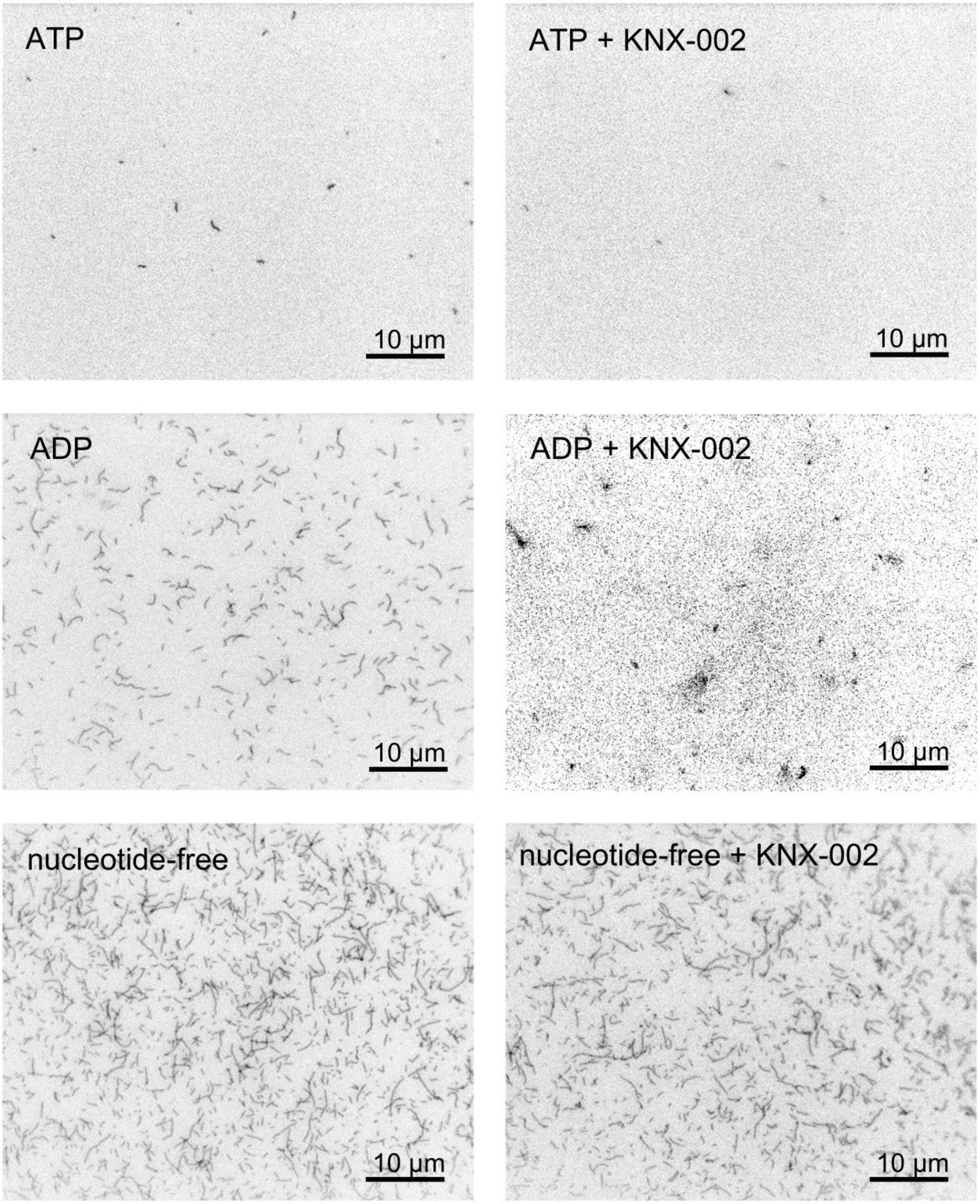

***Supplementary Figure 8** – Raw data showing examples of fields of actin bound to surface immobilized PfMyoA*. Direct comparison of the amount of actin bound in various nucleotides in the absence or presence of 100 µM KNX-002. Quantitation of the number of bound filaments is shown in **Fig. 5a** and **Supplementary Fig. 9**. Scale bar is 10 µm.

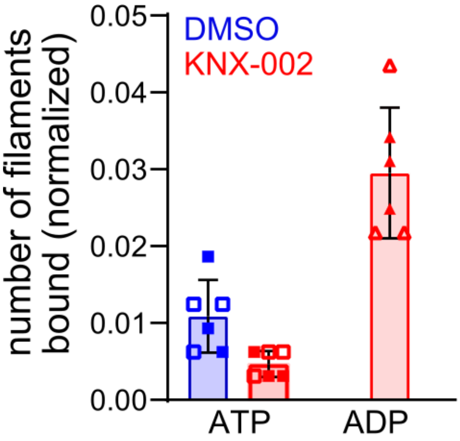

<u>Supplementary Figure 9</u> *– <u>Expanded y-axis for data in Fig. 5a</u>.* Number of actin filaments bound ± SD to surface immobilized PfMyoA (see Methods): ATP, 0.011 ± 0.004; ATP + KNX-002, 0.008 ± 0.030; ADP, 0.59 ± 0.05 (see **Fig. 5a**); ADP + KNX-002, 0.030 ± 0.008. Data represent two experiments each performed in triplicate with independent protein preparations (open and filled circles). No significant differences were observed between any pairs of the illustrated data: ATP ± KNX-002 ($p>0.999$), ADP+KNX-002 and ATP+KNX-002 ($p=0.971$), ADP+KNX-002 and ATP ($p=0.992$) (one-sided ANOVA followed by Tukey's post-hoc test). Conditions: 25 mM imidazole pH 7.5, 150 mM KCl, 4 mM $MgCl_2$, 1 mM EGTA, 10 mM DTT, 0% methylcellulose, 1% DMSO, 30°C. 2 mM MgATP or MgADP were added for +ATP or +ADP condition, respectively. Data bars are mean ± SD.

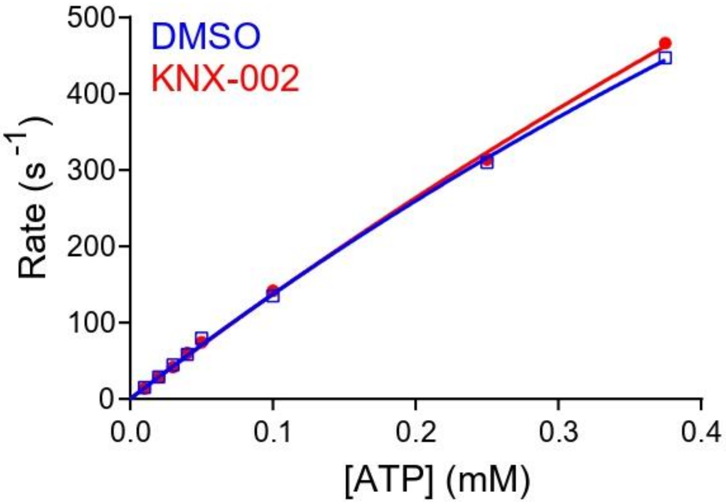

<u>Supplementary Figure 10</u> *– <u>KNX-002 does not affect the rate of ATP induced actomyosin dissociation</u>.* 0.3 µM PfMyoA and 0.4 µM skeletal actin were rapidly mixed with ATP in either 1% DMSO (blue open squares) or 100 µM KNX-002 (red filled circles). The apparent second order binding constant ± SD: 1% DMSO, 1.46 ± 0.14 μM^{-1} s^{-1}; KNX-002, 1.53 ± 0.33 μM^{-1} s^{-1}. Conditions: 10 mM HEPES pH 7.5, 50 mM KCl, 4 mM $MgCl_2$, 1 mM EGTA, 1% DMSO, 15°C. Source data are provided as a Source Data file.

Supplementary Figure 11 *– Uncropped gel image from Supplementary Figure 5a.* Lanes 2-4 are shown in Supplementary Figure 5a.

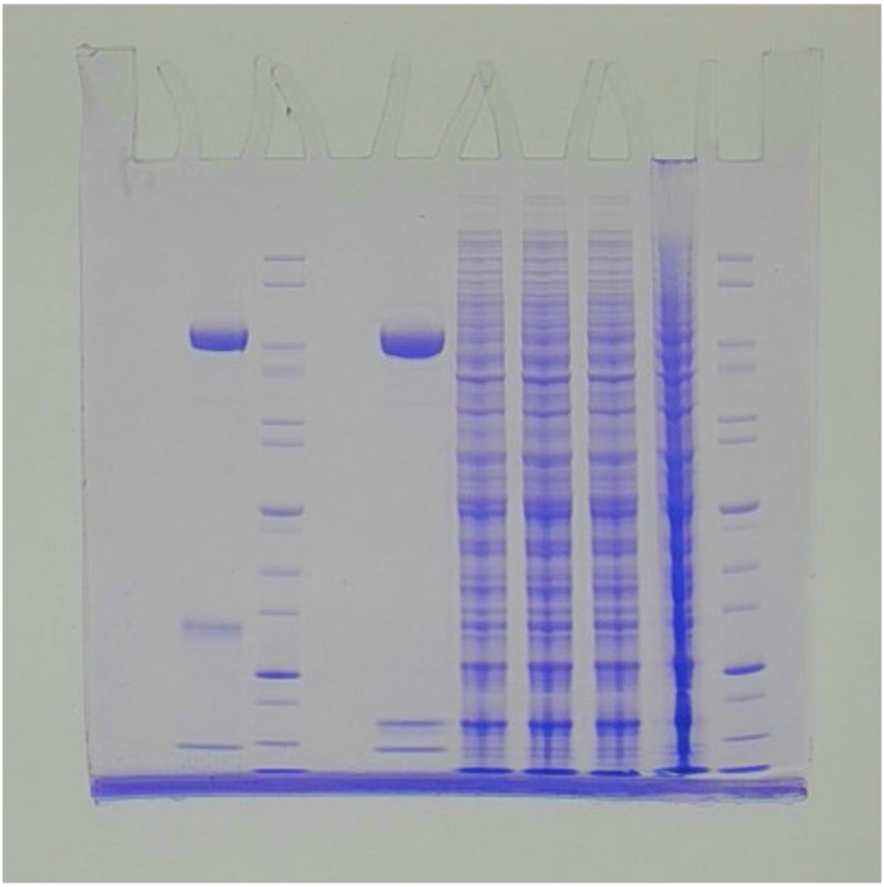

***Supplementary Table 1** – Data collection and refinement statistics (molecular replacement)*

	PfMyoA/ATPγS/Apo (8A12)	PfMyoA/ATPγS/KNX-002 (8CDQ)	PfMyoA/NF/KNX-002 (8CDM)
**Data collection**			
Space group	$P2_12_12_1$	$P2_12_12_1$	$P2_12_12_1$
Cell dimensions			
a, *b*, *c* (Å)	90.13, 115.14, 190.22	114.42, 170.92, 90.40	89.93, 114.41, 170.33
α, β, γ (°)	90, 90, 90	90, 90, 90	90, 90, 90
Resolution (Å)	85.11-2.03 (2.22-2.03)	95.08-2.21 (2.43-2.21)	94.97-2.36 (2.53-2.36)
R_{merge} (all I+ & I-)	0.078 (1.136)	0.199 (1.476)	0.144 (1.672)
R_{merge} (within I+ & I-)	0.073 (1.037)	0.189 (1.384)	0.140 (1.605)
Number of observations (total)	606008 (30283)	397437 (21446)	528650 (25814)
Number of observations (unique)	87835 (4371)	57115 (2857)	55344 (2767)
$<I / \sigma I>$	13.1 (1.6)	8.1 (1.5)	11.3 (1.5)
Completeness (Spherical) (%)	85.8 (15.9)	64.0 (13.2)	75.4 (19.9)
Completeness (Ellipsoidal) (%)	94.4 (63.0)	93.9 (59.2)	93.9 (69.2)
Redundancy	6.9 (6.9)	7.0 (7.5)	9.6 (9.7)
$CC_{1/2}$	0.999 (0.632)	0.992 (0.546)	0.997 (0.578)
**Refinement**			
Resolution (Å)	42.56-2.03 (2.10-2.03)	95.08-2.21 (2.29-2.21)	46.44-2.355 (2.44-2.36)
No. reflections	87338 (693)	57104 (274)	55334 (821)
R_{work} / R_{free}	0.1886/0.2324	0.1882/0.2353	0.1964/0.2482
No. atoms	9182	8998	8946
Protein	8641	8583	8546
Ligand/ion	46	110	44
Water	495	305	356
B-factors	58.18	50.08	58.72
Protein	58.38	40.37	58.84
Ligand/ion	49.71	43.14	48.64
Water	55.48	44.31	57.05
R.m.s. deviations			
Bond length (Å)	0.014	0.009	0.008
Bond angle (°)	1.21	0.98	0.92
PDB code	8A12		

*Number of xtals for each structure should be noted in footnote. *Values in parentheses are for highest-resolution shell.

***Supplementary Table 2** – Contacts between ligand and protein residues*

**PfMyoA**	*KNX-002 (8CDQ– this study)*	**Dd Myo2 (1YV3)**	*Blebbistatin*
$_{\text{Hw-h}}$**I641**	(D) Methoxyphenyl	$_{\text{Hw-h}}$**V630**	(D)
$_{\text{Hp-h}}$**E482**	(D)	$_{\text{Hp-h}}$**E467**	Phenyl (D)
$_{\text{Hp-h}}$**F485**	(D)	$_{\text{Hp-h}}$**C470**	
$_{\text{Hp-h}}$**L481**		$_{\text{Hp-h}}$**F466**	(D)
$_{\text{ϕLL-linker}}$**L272 mc**	(D) few interactions	$_{\text{ϕLL-linker}}$**L263 mc**	(D) few interactions
$_{\text{Hp-h}}$**I486**	(A,B,C,D)	$_{\text{Hp-h}}$**I471**	(C)
$_{\text{ϕLL-linker}}$**L271**	(A, C, D) (electrostatic)	$_{\text{ϕLL-linker}}$**L262**	(B,C,D) (electrostatic)
$_{\text{ϕLL-linker}}$**F270**	(A) Thiophene	$_{\text{ϕLL-linker}}$**Y261**	(A,B) (pi-stacking)
$_{\text{Hp-h}}$**T489**	(A)	$_{\text{Hp-h}}$**T474**	(A)
$_{\text{Hw-h}}$**F645**	(A)	$_{\text{Hw-h}}$**V634**	(A,B,D)
$_{\text{Hw-h}}$**Q648** $_{\text{Hw-h}}$**L649** $_{\text{Hw-h}}$**L652**		$_{\text{Hw-h}}$**Q637** $_{\text{Hw-h}}$**L638** $_{\text{Hw-h}}$**L641**	(A)
$_{\text{Sw2}}$**I470**	(A,B)	$_{\text{Sw2}}$**I455**	(A,B)
$_{\text{Sw2}}$**F471**	(B) (pi-stacking), (electrostatic) few A,C	$_{\text{Sw2}}$**S456**	(B,C) (electrostatic)
$_{\text{Sw1}}$**S246** $_{\text{Carbonyl}}$	(B) Pyrazole **Water-ATP-Mg^{2+}** (electrostatic)	$_{\text{Sw1}}$**S237** $_{\text{Carbonyl}}$	
$_{\text{Transd}}$**F248-G249 mc**	(B,C)	$_{\text{Transd}}$**F239-G240 mc**	(B,C) (electrostatic)
$_{\text{Sw1}}$**R247**	(C), few cyclopropamine	$_{\text{Sw1}}$**R238**	(C) few interactions
$_{\text{ϕLL-linker}}$**E273**	(C), few D	$_{\text{ϕLL-linker}}$**E264**	(D)

Blue background – sequences differ between PfMyoA and DdMyo2

Orange background – no interaction

***Supplementary Table 3** – Comparison of the binding modes of KNX-002 versus Blebbistatin*

Blebbistatin	KNX-002
**Differences**	
[a] Does not prevent ATP hydrolysis<br>Binds preferentially to ADP.Pi	Diminishes ATP hydrolysis (P_i burst experiment, Fig. 2d)
[a] Does not compete with nucleotide binding	Inhibits ATP binding 5-fold (Fig. 4c)
[a] Does not alter affinity for ADP at mM $MgCl_2$	KNX-002 decreases ADP affinity at 4 mM $MgCl_2$ (Fig. 4c)
[b] Does not weaken binding of M.ADP to A	Inhibits binding of M.ADP to A (Fig. 5a)
[a] Binds weakly to apoM (25 µM) compared to IC_{50} for ATPase activity (1.6 µM for basal/0.4 µM for actin activated)	Binds to nucleotide-free M (8.6 µM) and M.ADP (11.1 µM) with same order of magnitude as the IC_{50} ATPase activity (3.6 µM for basal/7.2 µM for actin- activated) (Supplementary Fig. 1 and Fig. 1a)
**Similarities**	
[a] Does not interfere with binding of M to A	Does not interfere with binding of M to A (Fig. 5a)
[a] Does not interfere with ATP induced dissociation of AM	Does not interfere with ATP induced dissociation of AM (Supplementary Fig. 10)
[a] Blocks myosin heads in a state that binds with low affinity to actin	Blocks myosin heads in a state that binds with low afffinity to actin (Fig. 5a)

[a]Kovacs, M. *et al*. J. Biol Chem 279:25557-63 (2004) [7]
[b]Takacs, B. *et al*. PNAS 107:6799-6804 (2010) [8]

Supplementary Table 4 *– KNX-002 pocket is conserved in sequence in Myosin A from different Apicomplexan genus.*

Myosin A sequences from different Apicomplexan genus are represented: *Plasmodium falciparum* (PfMyoA); *Toxoplasma gondii* (TgMyoA); *Eimeria tenella*; *Babesia bovis*; *Hepatocystis ssp*. All the residues involved in KNX-002 binding (in red) are conserved in all the MyoA, suggesting that KNX-002 would be able to inhibit all these motors. Fully conserved residues are labelled with an asterisk (*), residues conserved with groups of strongly similar properties are labelled with a colon (:), and residues conserved with groups of weakly similar properties are labelled with a period (.). The alignment was performed using ClustalOmega[9]. This conservation contrasts from the divergence seen when PfMyoA is aligned to human unconventional myosins (**Supplementary Fig. 4b**).

```
PfMyoA                    --MAVTNEEIKTASKIVRRVSNVEAFDKSGSVFKGYQIWTDISPTIENDPNIMFVKCVVQ 58
TgMyoA                    MASKTTSEELKTATALKKRSSDVHAVDHSGNVYKGFQIWTDLAPSVKEEPDLMFAKCIVQ 60
MyoA_Eimeria_tenella      ----MAAEEVKTAAGLKKKVSDVHVFDQSGAVFKGFQIWTDLAPAVREQPNLMFAKCAVQ 56
MyoA_Babesia_bovis        -MARFSKDEIATANALKRQGTAVQAFDASDNLLTGFQIWTDAAPSIKEDPSLLFVKCLVH 59
MyoA_Hepatocystis         --MAVTNEEIKTVSKIVKRASAIEAFDKSGGVFKGYQIWTDISPTIKENPNIMFVKCVVQ 58
                               : :*: *.  : :: : :...* *. : .*:***** :*::.::*.::*.** *:

PfMyoA                    QGSKKEKLTVVQIDPPG----TGTPYDIDPTHAWNCNSQVDPMSFGDIGLLNHTNIPCVL 114
TgMyoA                    AGTDKGNLTCVQIDPPG----FDEPFEVPQANAWNVNSLIDPMTYGDIGMLPHTNIPCVL 116
MyoA_Eimeria_tenella      SGSSKEVLVVQQVEPAG----GGETFEVPQAHAWNVNSAIDPMTYGDIGMLPHTNIPCVL 112
MyoA_Babesia_bovis        PGSTREKLICSQIEPAPPAGQEDKTFEVPLANAWNTNMSIDPMTYGDIGMLPQTNIPCVL 119
MyoA_Hepatocystis         QGSKQDKLTVVQIDPPG----LGTPYEIDSTHAWNCNSQIDPMSFGDIGSLNHTNIPCVL 114
                           *: :  *   *::*       .  :::  ::*** *  :***::**** * :*******

PfMyoA                    DFLKHRYLKNQIYTTAVPLIVAINPYKDLGNTTNEWIRRYRDTADHTKLPPHVFTCAREA 174
TgMyoA                    DFLKVRFMKNQIYTTADPLVVAINPFRDLGNTTLDWIVRYRDTFDLSKLAPHVFYTARRA 176
MyoA_Eimeria_tenella      DFLRHRYLRNQIYTCADPLVVAINPFKDLGNTTKEWICRYRDADDFTKLPPHVFYTARVA 172
MyoA_Babesia_bovis        DYLRHRFMNNQIYSTADPLLVAINPFKDLGNATDAVIREYKTAADSLKLAPHVFRTARMA 179
MyoA_Hepatocystis         DFLKQRYVKNQIYTTAAPLIVAINPYKDLGNTTKEWIRRYRDASDYTKLPPHVFTCARTA 174
                          *:*: *::.****: * **:*****::****:*   * .*: : *  ** ****  ** *

PfMyoA                    LSNLHGVNKSQTIIVSGESGAGKTEATKQIMRYFASSKSGNMDLRIQTAIMAANPVLEAF 234
TgMyoA                    LDNLHAVNKSQTIIVSGESGAGKTEATKQIMRYFAAAKTGSMDLRIQNAIMAANPVLEAF 236
MyoA_Eimeria_tenella      LDNLHGVNKSQTIIVSGESGAGKTEATKQVMRYFAAAKGGAMDMRIQNAIMAANPVLEAF 232
MyoA_Babesia_bovis        LENVYDYKYSQTIIVSGESGAGKTEATKQMMRYFAYSQKGETSGKIQTAIMAANPVLEAF 239
MyoA_Hepatocystis         LENLHGVNKSQTIIVSGESGAGKTEATKQIMRYFASSKSGNMDLRIQTAIMAANPVLEAF 234
                          *.*::  : ********************:***** :: *  . :**.************

PfMyoA                    GNAKTIRNNNSSRFGRFMQLVISHEGGIRYGSVVAFLLEKSRIITQDDNERSYHIFYQFL 294
TgMyoA                    GNAKTIRNNNSSRFGRFMQLDVGREGGIKFGSVVAFLLEKSRVLTQDEQERSYHIFYQMC 296
MyoA_Eimeria_tenella      GNAKTIRNNNSSRFGRFMQLDVAKEGGIRYGSVVAFLLEKSRVLTQDEQERSYHIFYQLC 292
MyoA_Babesia_bovis        GNAKTIRNNNSSRFGRFMQLDVSEEGGIRYGSVVAFLLEKSRIITQDSDERSYHIFYQFL 299
MyoA_Hepatocystis         GNAKTIRNNNSSRFGRFMQLIISHEGGIKNGSIVAFLLEKSRIISQDENERSYHIFYQFL 294
                          ******************** :..****: **:*********:::**.:*********:

PfMyoA                    KGANSTMKSKFGLKGVTEYKLLNPNSTEVSGVDDVKDFEEVIESLKNMELSESDIEVIFS 354
TgMyoA                    KGADAAMKERFHILPLSEYKYINPLCLDAPGIDDVAEFHEVCESFRSMNLTEDEVASVWS 356
MyoA_Eimeria_tenella      KGADKAMRDKFHLLSLDQYTYINPKCFDVPGIDDIADYNDVCAAFKSMAMSEEQVEAVWS 352
MyoA_Babesia_bovis        KGATPEMRNKYKLKGLNEYKFLNPNCLDVPSIDDVADFKEVLESFKNMGLGESEIDTILS 359
MyoA_Hepatocystis         KGASSAMKSKYGLKDLKSYSLLNPNSVNVNGIDDVKDFNEVMESLKNMQLSDDQIDVIFS 354
                          ***   *:.:: :  : .*. :** . :. .:**: ::.:*  :::.* : :.::  : *

PfMyoA                    IVAGILTLGNVRLIEKQEAGLSDAAAIMDEDMGVFNKACELMYLDPELIKREILIKVTVA 414
TgMyoA                    IVSGVLLLGNVEVTATKDGGIDDAAAIEGKNLEVFKKACGLLFLDAERIREELTVKVSYA 416
MyoA_Eimeria_tenella      IVSGVLLLGNVKVTASKQGGIDDAAAIEGDNLTLFNQACQLLHIDAEAVLRELTLKVTYA 412
MyoA_Babesia_bovis        VLSGVLLMGNVEIGATSVDGIDDAAMIVNK--DVFNTACNLLYLDPALLETELTVKITSA 417
MyoA_Hepatocystis         IIAGILTLGNVRLIEVADAGLTDAAAIHNEDTGLFRKACELMFLDAEAVKREILIKVTIA 414
                          :::*:* :***.:      *: *** * ..   :*. ** *:.:*   :  *: :*:: *

PfMyoA                    GGTKIEGRWNKNDAEVLKSSLCKAMYEKLFLWIIRHLNSRIEPEGGFKTFMGMLDIFGFE 474
TgMyoA                    GNQEIRGRWKQEDGDMLKSSLAKAMYDKLFMWIIAVLNRSIKPPGGFKIFMGMLDIFGFE 476
MyoA_Eimeria_tenella      GGQKVESRWKQEDGDMLKSSLAKAMYDKLFLWIIRELNKSIEPPEGFRNFLGMLDIFGFE 472
MyoA_Babesia_bovis        GNNQIKGPRKKQDGEVLKTSVCKAIYERLFLWLIRNLNRSIEPPTGFKTFIGMLDIFGFE 477
MyoA_Hepatocystis         GGNKIEGRWNKKDAEVLKSSLCKAMYEKLFIWIINYLNSRIEPKDGFSVFMGMLDIFGFE 474
                          *. ::..  :::*.::**:*:.**:*::**:*:*  **  *:*  **  *:*********

PfMyoA                    VFKNNSLEQLFINITNEMLQKNFVDIVFERESKLYKDEGISTAELKYTSNKEVINVLCEK 534
TgMyoA                    VFKNNSLEQFFINITNEMLQKNFVDIVFDRESKLYRDEGVSSKELIFTSNAEVIKILTAK 536
MyoA_Eimeria_tenella      VFKNNSLEQFFINVTNEMLQKNFVDIVFTREAKLYKEEGVSTAELVYTSNADVISLLTDK 532
MyoA_Babesia_bovis        VFKNNSLEQLFINITNEMLQTNFVDIVFKREAALYTNEGIPIPELKYTSNADIIEVLCSK 537
MyoA_Hepatocystis         VFKNNSLEQLFINITNEMLQKNFIDIVFERETKLYREEGISSAELKYTSNKDVISVLCDK 534
                          *********:***:******.**:**** **: ** :**:   ** :*** ::*.:*  *

PfMyoA                    GKSVLSYLEDQCLAPGGTDEKFVSSCATNLKENNKFTPAKVASNKNFIIQHTIGPIQYCA 594
TgMyoA                    NNSVLAALEDQCLAPGGSDEKFLSTCKNALKGTTKFKPAKVSPNINFLISHTVGDIQYNA 596
MyoA_Eimeria_tenella      KSSILSTLEDQCLAPGGGDEKFVAACKSAFKNSPKFKPAKVSPNINFLVCHTIGDIQYNA 592
MyoA_Babesia_bovis        GKSILSLLEDQCLAPGGTDENLVSSCNNQLKDNSKYSPAKVGAKLNFTVTHTIGTISYNA 597
MyoA_Hepatocystis         GRSVLSYLEDQCLAPGGTDEKFVSACVINLKTNEKFIPAKVASNKNFLIQHTIGAIQYCA 594
                            *:*: ********** **:::::*   :* . *: ****. : ** : **:* *.* *
```

```
PfMyoA                        ESFLLKNKDVLRGDLVEVIKDSPNPIVQQLFEGQVIEKGKIAKGSLIGSQFLNQLTSLMN 654
TgMyoA                        EGFLFKNKDVLRAEIMEIVQQSKNPVVAQLFAGIVMEKGKMAKGQLIGSQFLSQLQSLME 656
MyoA_Eimeria_tenella          EGFLFKNKDVLRAEIMEVVQASPNPVVRDLFAGIVMEKGKIAKGQLIGSQFLRQLEALMD 652
MyoA_Babesia_bovis            QSFIFKNKDVLTAELVECVNASPNEMIKGLFENVVVTRGKLAKGQLIGSQFMSQLDALMK 657
MyoA_Hepatocystis             ENFLFKNKDVLRGELVEVIMASNNDIVKALFEGQIIEKGKIAKGSLIGSQFLNQLISLMS 654
                              :.*::****** .:::* :  * * ::  ** . :: :**:***.******: ** :**.

PfMyoA                        LINSTEPHFIRCIKPNENKKPLEWCEPKILIQLHALSILEALVLRQLGYSYRRTFEEFLY 714
TgMyoA                        LINSTEPHFIRCIKPNDTKKPLDWVPSKMLIQLHALSVLEALQLRQLGYSYRRPFKEFLF 716
MyoA_Eimeria_tenella          LINSTEPHFVRCVKPNDTKKPLDWVQSKVLIQLHALSVLEALQLRQVGFSYRRPFKDFLY 712
MyoA_Babesia_bovis            LINSTDSHFIRCVKPNESKQPLDFNSVKILVQLHALSIIEALQLRKLGYSYRRPYESFLE 717
MyoA_Hepatocystis             LINCTEPHFIRCIKPNENKKPLEWCEPKILIQLHALSILEALTLRQLGYSYRRTFKEFLY 714
                              ***.*: **:**:***:.*:**::   *:*:******::*** **::*:**** ::.**

PfMyoA                        QYKFVDIAAAEDSSVENQNKCVNILKLSGLSESMYKIGKSMVFLKQEGAKILTKIQREKL 774
TgMyoA                        QFKFIDLSASENPNLDPKEAALRLLKSSKLPSEEYQLGKTMVFLKQTGAKELTQIQRECL 776
MyoA_Eimeria_tenella          QFKFIDLGICENPNLSPKEACEALLEKAKVSKSGCQVGKTMIFLKQDAAKQLTLLQRQCL 772
MyoA_Babesia_bovis            QYKYLDLSITNDKSLDPKVAAERLITSVGVSKDQYAFGKSMMFLTQKLAKELTAIQRQRL 777
MyoA_Hepatocystis             QYKFVDIAVTEDSSIDDANKCVKILKNSGLSENMYKLGKTMVFLKQEGAKALTKYQREKL 774
                              *:*::*:.  :: .:.    .  ::    : ..   .**:*:**.*  ** **  **: *

PfMyoA                        VEWENCVSVIEAAILKHKYKQKVNKNIPSLLRVQAHIRKKMVAQ---------------- 818
TgMyoA                        SSWEPLVSVLEAYYAGRRHKKQLLKKTPFIIRAQAHIRRHLVDNNVSPATVQPAF----- 831
MyoA_Eimeria_tenella          AAWTPVVNVLEACYLKLRLKKAIQKKQHYIIRTQAHIRRHIVDHNVSPPAITKLF----- 827
MyoA_Babesia_bovis            SVWEPITAMIDAWITRQMAKKSLMSAVEDIVRLQAHLRRHIVESRVTPSPDRMVKMQMTS 837
MyoA_Hepatocystis             VEWEKCIGVIEAAVIKQRYRKNINEIIPSLVRVQAHIKKRLMA----------------- 817
                                *     :::*       :: : .    ::* ***::::::
```

***Supplementary Table 5** – Key Resources*

REAGENT or RESOURCE	Symbol/name used in publication	SOURCE	IDENTIFIER
**Chemicals**			
Adenosine 5'-triphosphate disodium salt (Grade I)	ATP	Sigma	A2383
Adenosine 5'-diphosphate sodium salt	ADP	Sigma	A2754
Anti-FLAG M2 Affinity Gel	FLAG resin	Sigma	A2220
Biotinylated bovine serum albumin	B-BSA	Thermo Scientific	29130
Pyruvate Kinase from rabbit muscle	pyruvate kinase	Sigma	P9136
Phospho(enol)pyruvic acid monosodium salt hydrate	phosphoenolpyruvate	Sigma	P0564
L-lactic dehydrogenase from rabbit muscle	L-lactate dehydrogenase	Sigma	L1254
β-Nicotinamide adenine dinucleotide, reduced disodium salt	NADH	Sigma	N9410
NeutrAvidin	neutravidin	Thermo Scientific	31000
Catalase from bovine liver	catalase	Sigma	C40
Glucose oxidase nfrom Aspergillus niger	glucose oxidase	Sigma	G6125
Malachite green	malachite green	Fisher	M-290
**Recombinant DNA**			
Plasmodium myosin A	PfMyoA	(Bookwalter *et al.*, 2017)[10]	NCBI:XM_001350111.1
Plasmodium falciparum microtubule interacting protein	PfMTIP	(Bookwalter *et al.*, 2017)[10]	NCBI:XM_001350813.1
Plasmodium falciparum essential light chain	PfELC	(Bookwalter *et al.*, 2017)[10]	NCBI:XM_001347419.1
Plasmodium UCS family chaperone	PUNC	(Bookwalter *et al.*, 2017)[10]	chimeric clone of NCBI:XM_001348333.1 and NCBI:XM_002260772
**Proteins**			
Plasmodium falciparum myosin A (wild-type)	WT	(Robert-Paganin *et al.*, 2019)[11]	
Plasmodium falciparum myosin A (F270Y/F471A/F645H)	F/YAH	this paper	
actin	actin	chicken breast	
Rhodamine-phalloidin	labelled actin	Invitrogen	R415
FLAG peptide	FLAG peptide	APExBIO	A6002

**Imaging and Data Analysis**			
Lambda 25 UV/VIS spectrophotometer		Perkin Elmer	
Zeiss Axiovert 10 microscope		Zeiss	
Rolera Mgi Plus digital camera		Qimaging	
Nikon NIS Elements software		Nikon	v3.22.15
KinTec SF-2001 stopped-flow apparatus		KinTec	
Prism		GraphPad	v9.3.1; RRID:SCR_002798
Fast Automated Spud Trekker	FAST	available for free download at spudlab.stanford.edu, also see [12]	v1.1

Supplementary references

1. Llinas, P. *et al.* How Actin Initiates the Motor Activity of Myosin. *Dev. Cell* **33**, 401–412 (2015).
2. Robert-Paganin, J., Pylypenko, O., Kikuti, C., Sweeney, H. L. & Houdusse, A. Force Generation by Myosin Motors: A Structural Perspective. *Chem. Rev.* **120**, 5–35 (2020).
3. Moussaoui, D. *et al.* Full-length Plasmodium falciparum myosin A and essential light chain PfELC structures provide new anti-malarial targets. *Elife* **9**, (2020).
4. Robert-Paganin, J., Auguin, D. & Houdusse, A. Hypertrophic cardiomyopathy disease results from disparate impairments of cardiac myosin function and auto-inhibition. *Nat. Commun.* **9**, 4019 (2018).
5. Gulick, A. M., Bauer, C. B., Thoden, J. B. & Rayment, I. X-ray Structures of the MgADP, MgATPγS, and MgAMPPNP Complexes of the Dictyostelium discoideum Myosin Motor Domain,. *Biochemistry* **36**, 11619–11628 (1997).
6. Bauer, C. B., Holden, H. M., Thoden, J. B., Smith, R. & Rayment, I. X-ray structures of the apo and MgATP-bound states of Dictyostelium discoideum myosin motor domain. *J. Biol. Chem.* **275**, 38494–38499 (2000).
7. Kovács, M., Tóth, J., Hetényi, C., Málnási-Csizmadia, A. & Sellers, J. R. Mechanism of blebbistatin inhibition of myosin II. *J. Biol. Chem.* **279**, 35557–35563 (2004).
8. Takács, B. *et al.* Myosin complexed with ADP and blebbistatin reversibly adopts a conformation resembling the start point of the working stroke. *Proc. Natl. Acad. Sci. U. S. A.* **107**, 6799–6804 (2010).
9. McWilliam, H. *et al.* Analysis Tool Web Services from the EMBL-EBI. *Nucleic Acids Res.* **41**, W597-600 (2013).
10. Lu, H., Fagnant, P. M. & Trybus, K. M. Unusual dynamics of the divergent malaria parasite PfAct1 actin filament. *Proc. Natl. Acad. Sci. U. S. A.* **116**, 20418–20427 (2019).
11. Robert-Paganin, J. *et al.* Plasmodium myosin A drives parasite invasion by an atypical force generating mechanism. *Nat. Commun.* **10**, 3286 (2019).
12. Aksel, T., Choe Yu, E., Sutton, S., Ruppel, K. M. & Spudich, J. A. Ensemble force changes that result from human cardiac myosin mutations and a small-molecule effector. *Cell Rep.* **11**, 910–920 (2015).
