# Supplementary material for: Mechanism of small molecule inhibition of Plasmodium falciparum myosin A informs antimalarial drug design
Source: Nat Commun. 2023 Jun 12;14:3463. doi: 10.1038/s41467-023-38976-7 (PMC10261046; doi:10.1038/s41467-023-38976-7)
Supplement: Supplementary file 3 — Description of Additional Supplementary Files [file 41467_2023_38976_MOESM3_ESM.pdf]

Description of Additional Supplementary Files

File Name: Supplementary Movie 1

Description: Movement of actin filaments by PfMyoA in the absence of KNX-002.

Representative movie showing directed motion of actin filaments powered by PfMyoA. Conditions: 25 mM imidazole pH 7.5, 150 mM KCl, 4 mM $MgCl_2$, 1 mM EGTA, 10 mM DTT, 0.15% methylcellulose, 1% DMSO, 30°C.

File Name: Supplementary Movie 2

Description: In the presence of KNX-002, no directed movement of actin filaments by PfMyoA is observed.

In the presence of 200 μM KNX-002, few filaments are seen in the field of view and none exhibit directed motion. Note that the presence of KNX-002 causes photobleaching of the rhodamine-phalloidin labeled actin filaments, resulting in grainier images. Conditions: 25 mM imidazole pH 7.5, 150 mM KCl, 4 mM $MgCl_2$, 1 mM EGTA, 10 mM DTT, 0.15% methylcellulose, 1% DMSO, 30°C.

File Name: Supplementary Movie 3

Description: KNX-002 and Blebbistatin (Blebb) target different pockets.

Comparison of the binding site of KNX-002 and Blebb using structures (PfMyoA-KNX-002-MgATPγS and PDB code 1YV3) aligned on the U50 subdomain (blue) of the motor domain. The movie starts with an overall view of myosin comparing PfMyoA with KNX-002 bound and Dictyostelium discoideum myosin 2 (DdMyo2) complexed to Blebb. KNX-002 is yellow and Blebb is green. Note that the Converter (green subdomain) rotates in this animation as the two myosins are not in the same conformational state: PfMyoA is in the post-rigor state when bound to KNX-002, while Myo2 is in the Pre-powerstroke state when bound to Blebb. Conformational changes during the recovery stroke allow repriming of the motor between

these two ATP-bound states when myosin is unbound to inhibitors. Note that the inner cleft where the two drugs bind differ in conformation due to a repositioning of Switch-2 (orange) during the transition. The second part of the movie shows a zoom on the binding pocket of KNX-002 and displays alternatively the Blebb binding pocket. It allows one to appreciate the change in the Switch-2 conformation, as well as the difference in position of the residues involved in binding of these inhibitors (spheres). In red are residues of the HW helix involved in Blebb binding but that are not part of the KNX-002 binding site.
